# Sentinel surveillance and epidemiology of *Clostridioides difficile* in Denmark, 2016 to 2019

**DOI:** 10.2807/1560-7917.ES.2022.27.49.2200244

**Published:** 2022-12-08

**Authors:** Søren Persson, Hans Linde Nielsen, John Eugenio Coia, Jørgen Engberg, Bente Scharvik Olesen, Anne Line Engsbro, Andreas Munk Petersen, Hanne Marie Holt, Lars Lemming, Ea Sofie Marmolin, Turid Snekloth Søndergaard, Leif Percival Andersen, Mie Birgitte Frid Jensen, Camilla Wiuff, Gitte Sørensen, Sofie Holtsmark Nielsen, Eva Møller Nielsen

**Affiliations:** 1 Department of Bacteria, Parasites and Fungi, Statens Serum Institut, Copenhagen, Denmark; 2 Department of Clinical Microbiology, Aalborg University Hospital, Aalborg, Denmark; 3 Department of Clinical Medicine, Aalborg University, Aalborg, Denmark; 4 Department of Clinical Microbiology, Esbjerg Hospital, University of Southern Denmark, Esbjerg, Denmark; 5 Department of Regional Health Research IRS, University of Southern Denmark, Esbjerg, Denmark; 6 Department of Clinical Microbiology, Zealand University Hospital, Køge, Denmark; 7 Department of Clinical Microbiology, Copenhagen University Hospital – Herlev and Gentofte, Herlev, Denmark; 8 Department of Clinical Microbiology, Copenhagen University Hospital Hvidovre, Hvidovre, Denmark; 9 Department of Gastroenterology, Copenhagen University Hospital Hvidovre, Hvidovre, Denmark; 10 Department of Clinical Microbiology, Odense University Hospital, Odense, Denmark; 11 Department of Clinical Microbiology, Aarhus University Hospital, Aarhus, Denmark; 12 Department of Clinical Microbiology, Vejle Hospital, Vejle, Denmark; 13 Department of Clinical Microbiology, Sønderjylland Hospital, Aabenraa, Denmark; 14 Department of Clinical Microbiology, Copenhagen University Hospital (Rigshospitalet), Copenhagen, Denmark

**Keywords:** Clostridioides difficile, typing, epidemiology, surveillance

## Abstract

**Background:**

Since 2008, Danish national surveillance of *Clostridioides difficile* has focused on binary toxin-positive strains in order to monitor epidemic types such as PCR ribotype (RT) 027 and 078. Additional surveillance is needed to provide a more unbiased representation of all strains from the clinical reservoir.

**Aim:**

Setting up a new sentinel surveillance scheme for an improved understanding of type distribution relative to time, geography and epidemiology, here presenting data from 2016 to 2019.

**Methods:**

For 2─4 weeks in spring and autumn each year between 2016 and 2019, all 10 Danish Departments of Clinical Microbiology collected faecal samples containing toxigenic *C. difficile*. Isolates were typed at the national reference laboratory at Statens Serum Institut. The typing method in 2016–17 used tandem-repeat-sequence typing, while the typing method in 2018–19 was whole genome sequencing.

**Results:**

During the study period, the sentinel surveillance scheme included ca 14–15% of all Danish cases of *C. difficile* infections. Binary toxin-negative strains accounted for 75% and 16 of the 20 most prevalent types. The most common sequence types (ST) were ST2/13 (RT014/020) (19.5%), ST1 (RT027) (10.8%), ST11 (RT078) (6.7%), ST8 (RT002) (6.6%) and ST6 (RT005/117) (5.1%). The data also highlighted geographical differences, mostly related to ST1 and temporal decline of ST1 (p = 0.0008) and the increase of ST103 (p = 0.002), ST17 (p = 0.004) and ST37 (p = 0.003), the latter three binary toxin-negative.

**Conclusion:**

Sentinel surveillance allowed nationwide monitoring of geographical differences and temporal changes in *C. difficile* infections in Denmark, including emerging types, regardless of binary toxin status.

**Key public health message**

**What did you want to address in this study?**

Before 2016, national surveillance focused on the major *Clostridioides difficile* outbreak strains and severe outcome patients. While this setup is essential for hospitals to react to outbreaks, we are unable to monitor all clinically relevant strains. We wanted to know if the use of an additional sentinel surveillance that included a subset of toxigenic strains from Danish clinical microbiology departments would allow us to obtain more comprehensive information.

**What have we learnt from this study?**

We found that strains without the binary toxin are dominating and increasing in prevalence, while other strains, in particular ST1/RT027 which can cause severe disease, differ greatly in geographical distribution.

**What are the implications of your findings for public health?**

It is possible to monitor the changing epidemiology of *C. difficile* by investigating a relatively small fraction of national cases (ca 15%) in a well-organised setup. This sentinel surveillance has been shown to be an informative and relatively inexpensive complement to national surveillance, and has now been implemented as a routine setup, supplementing information on outbreak strains obtained by the continuous national surveillance.

## Introduction

*Clostridioides difficile* infection (CDI) is the leading cause of antibiotic-associated diarrhoea and a common nosocomial pathogen, often leading to severe and recurrent infections [1]. Since the beginning of the millennium, several large hospital outbreaks have been identified, mostly in the United States (US) and Europe, associated with the global spread of the PCR ribotype (RT) 027 (multi locus sequence type 1) (ST1) as the predominant type [2-4]. A number of other types have also been implicated as epidemic strains [5] and studies have observed an increasing number of community acquired CDIs of possible zoonotic origin [6,7]. The first registered outbreak of ST1(RT027) in Denmark was a small outbreak in the western part of the country between 2006 and 2007 [8], and soon thereafter, larger outbreaks were identified at major hospitals in the eastern part of Denmark [9].

In Denmark, departments of clinical microbiology (DCMs) located at 10 major hospitals, perform the primary diagnostics and local surveillance of all *C. difficile* cases. In order to monitor the national epidemiology of ST1(RT027) and other binary toxin-positive strains, guidelines were issued in 2008 by the Danish Health Authority for DCMs to submit faecal samples or bacterial isolates to the national reference laboratory at Statens Serum Institut (SSI), Copenhagen under the following criteria: (i) binary toxin-positive; (ii) severe clinical manifestations; (iii) part of an outbreak (iv) moxifloxacin resistance. These positive samples then underwent further typing.

Since several reports have shown that strains without the binary toxin are important in terms of disease burden, prevalence, increasing community acquired CDI, multidrug resistance (MDR) and epidemic potential [10-12], a sentinel surveillance was initiated in 2016 as an addition to the existing surveillance. The sentinel surveillance is a compromise between cost and a representativeness of cases. It involves all DCMs submitting all toxigenic isolates or faecal samples (minimum positive for gene(s) encoding *C. difficile* toxin A (*tcdA*) and/or B (*tcdB*) for typing at SSI during a 2–4 weeks period in both the spring and the autumn. Here, we present the data from 4 years (2016–2019) of sentinel surveillance of *C. difficile*.

## Methods

### Settings, inclusion criteria and diagnostics

The Danish healthcare system, where all citizens have free access to public healthcare, operates across three political and administrative levels: the state, the regions and the municipalities. The five geographical regions: North Denmark Region (containing 1 DCM), Central Denmark Region (containing 1 DCM), Region of Southern Denmark (containing 4 DCMs), Region Zealand (containing 1 DCM) and Capital Region of Denmark (containing 3 DCMs) are responsible for the hospital care and healthcare services provided by general practitioners. The DCMs receive samples from hospitals and general practitioners. However, some local differences exist regarding patient inclusion criteria and diagnostic methods. Briefly, stool samples are collected from patients suspected of CDI based on clinical presentation. Some DCMs use additional criteria including age (i.e. patients ≥ 65 years), hospitalisation and antibiotic exposure. For primary diagnostic methods, seven DCMs use direct PCR, either in-house or commercial or a combination, two DCMs use culture and one uses PCR and culture on samples from hospitals and general practitioners, respectively (see Supplementary Table S1). Eight DCMs collect samples in containers without a buffer and two DCMs used FecalSwab (COPAN ITALIA, Brescia, Italy).

Samples for the sentinel surveillance were collected every year from 2016 to 2019 during spring (February to May) and autumn (October to November) in periods of either 2 weeks or 1 month (Table 1). The European Centre for Disease Prevention and Control (ECDC) case definition was applied [13], i.e. only the first sample was included if an identical type was identified from the same patient within 8 weeks of the first sample, but as a new case if a different type was identified. If the sampling time was more than 8 weeks apart, the same patient was included again as a new case. Children under 2 years were excluded from the general dataset, and their samples were analysed separately. Samples received at SSI were processed within 24 hours. From faecal samples and faecal swabs (with enough material to colour the buffer), 10 µL sterile loop of material was transferred to 2mL sterile buffered saline. From faecal swabs with less material, the stick was transferred and briefly shaken in 2 mL sterile buffered saline. The saline suspension was heated at 62 °C for 10 min and from there a 1 µL sterile loop was streaked out on a ChromID *C. difficile* agar plate (bioMérieux, Craponne, France) and incubated at 37 °C for 24 hours under anaerobic conditions. One single colony was subcultured on a 5% blood agar plate (SSI Diagnostica, Hillerød, Denmark) for 24 hours under anaerobic conditions and identification of *C. difficile* was confirmed by matrix-assisted laser desorption/ionisation time-of-flight (MALDI-TOF) mass spectrometry (Microflex LRF, Bruker, Billerica, US) using flexControl software version 3.1.65–4.1.80 with the library Compass for flexSeries 1.4 (Bruker, Billerica, US). Culturing was attempted twice and if unsuccessful, the sample was classified as non-cultivable.

**Table 1:** Characteristics of the *Clostidioides difficile* sentinel surveillance set up and patients included, by year, Denmark, 2016–2019

Characteristic	2016		2017		2018		2019	
Surveillance set up and results								
Duration (spring and autumn)	2x2 weeks		2x1 month		2x1 month		2x1 month	
Typing method	TRST		TRST		WGS/ST		WGS/ST	
Toxin gene detection	PCR		PCR		WGS script		WGS script	
Unique types/non-typable	54/6		75/22		77/7		76/9	
%*cdtAB* negative	70.0		65.6		77.1		79.5	
Simpson’s diversity index	0.89		0.90		0.92		0.93	
Total number of samples (n = 2,692); unique patients (n = 2,594)^a^								
Age group (years)	n	%	n	%	n	%	n	%
2–17	9	4.6	14	1.8	22	2.8	18	2.6
18–44	51	11.7	84	10.8	76	9.8	82	11.7
45–64	90	20.6	132	17.0	140	18.0	120	17.1
≥ 65	286	65.6	548	70.4	539	69.4	481	68.6
Female	%	p value	%	p value	%	p value	%	p value
	54.6	0.06	53.9	0.03	58.2	0.000003	54.2	0.03
Age in years and number of patients	n	Median age (range)	n	Median age (range)	n	Median age (range)	n	Median age (range)
Females	238	74 (3–95)	419	74(2–99)	452	73(2–97)	380	73 (3–103)
Males	198	70 (2–95)	359	73 (2–97)	325	72(2–95)	321	72 (2–94)
All	436	72 (2–95)	778	73 (2–99)	777	73(2–97)	701	73(2–103)

*cdtAB*: genes encoding *C. difficile* binary toxin; MiBa: Danish Microbiological Database; ST: sequence type, TRST: tandem repeat sequence type, WGS: whole genome sequencing.

^a^ All presented age distributions are based on total number of samples, i.e. some patients participated more than once (see Results).

Spring data collection took place between February and May and autumn data collection took place between October and November.

### Typing methods

In 2016 and 2017, DNA from bacterial colonies was extracted by Qiagen’s Blood and Tissue kit (Qiagen, Hilden, Germany) with a prelysis containing 1.2% Triton X-100 and 20mg/mL lysozyme for 1 hour at 37 °C. Typing was performed by tandem repeat sequence typing (TRST) according to Zaiss et al. [14], and toxin profiling was performed by multiplex PCR [15]. In 2018 and 2019, isolates underwent whole genome sequencing (WGS) using the following procedure: colonies were lysed using proteinase K plus lysozyme and lysostaphin followed by purification of genomic DNA using the DNA and Viral NA small volume kit on the MagNa Pure 96 system (Roche Diagnostics, Rotkreuz, Switzerland). DNA was diluted to 0.2–0.5 ng/µl using Quant-iT-dsDNA BR and HS assays (ThermoFisher Scientific, Waltham, US). Samples were prepared for Next Generation Sequencing on the Microlab STAR line liquid handling workstation from Hamilton (Hamilton, Reno, US), using Illumina Nextera XT library preparation (Illumina, San Diego, US). Samples (post library concentration ≥ 1 ng/µl) were pooled and normalised according to genome size and subsequently sequenced on the Illumina NextSeq platform (mid-out 2x150 bp cycles). Quality control of the data was performed using an in-house QC pipeline (https://github.com/ssi-dk/bifrost) before further downstream analyses. The publicly available database pubmlst.org [16] was applied and run with a local script, to determine sequence type (ST) directly from WGS data. BioNumerics version 7.6.3 (bioMérieux) was used to construct core genome multilocus sequence typing (cgMLST) phylogeny based on the analysis of 1,999 genes (Bionumerics scheme). When possible, PCR ribotypes were inferred from MLST and by phylogenetic comparison to reference strains in our local cgMLST database. Toxin profiling was part of the local script (Supplementary Table S2) mentioned above, using 90% identity as limit for positive toxin identification.

### Statistics

All statistical analysis was performed using R version 4.1.2 [17]. Simpson diversity index was calculated for each year and region using the diversity function (vegan version 2.5-7). The Simpson index was based on D = sum p_i^2, where p_i is the proportional abundance of type i, which returns 1-D. Binomial regression was used to test whether the STs significantly increased or decreased over the 4 years. To test for significant age difference between ST1 and ST11(RT078), a two-sided Wilcoxon test was performed. To test for significant difference in sex, a two-sided chi-squared test was performed. Significance level of p<0.01 was used.

## Results

A total of 3,458 samples were collected between 2016 and 2019. Among these samples, 766 were excluded due to the following reasons: 295 could not be cultured, 85 were non-toxigenic (Supplementary Table S3), 198 were doublet samples, i.e. same patient, type and sample date, 112 were doublet samples from the same patient with the same type less than 8 weeks apart (the first sample was included) and 76 samples were from patients under 2 years of age (Supplementary Table S4). This left a dataset of 2,692 samples for further analysis (Supplementary Table S5).

The data presented here represents 4 years of sentinel surveillance, i.e. two periods of 2 weeks in 2016 and two periods of 1 month duration in 2017, 2018 and 2019 (Table 1). Complete nationwide *C. difficile* cases were available from the Danish Microbiological Database (MiBa) for 2018 (5,173) and 2019 (4,920), indicating that the sentinel surveillance made up ca 15% (2018) and 14% (2019) of all national diagnosed cases, with incidences of 89 per 100,000 and 85 per 100,000 inhabitants for 2018 and 2019, respectively. For the complete dataset, the median age was 73 years and 55.5% were females (p = 0.00000002). There were more females than males in each of the 4 years, the largest difference being seen in 2018 (see Table 1).

### Analysis related to typing

ST was inferred from TRST in 2016–17 and directly from WGS in 2018–19. Table 2 shows the overall association between the two methods including corresponding RT and clades of the 20 major *C. difficile* types. ST2/13 was the most common type (19.5%) followed by ST1 (10.8%), ST11(RT078) (6.7%) and ST8 (6.6%). To correlate ST with the two different methods (TRST and RT) and because of different typing resolution within these methods, some STs reported here had to be expanded to include several types, i.e. RT014/020, RT014 and RT012 are reported as ST2/13, ST14/49 and ST21/54/139, respectively. ST11 contains two distinct main types not resolved by MLST, namely RT066 (TR067) and RT078 (TR070), both identified from WGS by cgMLST phylogeny and are here referred to as ST11(RT066) and ST11(RT078), respectively. Median age and percentage females were calculated for each ST (Table 2). We did not make direct comparisons regarding age distribution between all STs, but patients who tested positive for ST1 (median age 77 years) were older than patients who tested positive for ST11(RT078) (median age 71.5 years) (p = 0.00003).

**Table 2:** The 20 most common *Clostridioides difficile* strains in descending order of prevalence, and association between sequence type (ST), tandem repeat sequence type (TRST), PCR ribotype (RT), clade, presence of binary toxin (*cdtAB*) and patient characteristics, Denmark, 2016–2019

ST	TRST	RT	Clade	*cdtAB*	Samples		Median age of patient in years (range)	Females	
					n	%		n	%
2/13[102/110]	014[117/241]	014/020	1	Neg	525	19.5	71 (2–97)	302	57.5
1	027/210	027	2	Pos	290	10.8	77 (22–97)	163	56.2
11(RT078)	070	078	5	Pos	180	6.7	71.5 (2–97)	103	57.2
8	002/115	002	1	Neg	179	6.6	74 (3–95)	107	60
6	051/052[042]	005/117	1	Neg	138	5.1	76 (2–96)	70	50.1
14/49	065[238/240/294]	014	1	Neg	134	5	69 (7–97)	69	51.5
21/54/139	012	012	1	Neg	99	3.7	74 (9–97)	54	54.5
3	001/072	001	1	Neg	89	3.3	73 (7–99)	46	51.7
103	043	043	1	Neg	80	3	77 (19–103)	41	51.3
5	016/092/094	023	3	Pos	68	2.5	72 (4–91)	36	52.9
55	048[211]	070	1	Neg	67	2.5	73 (2–95)	31	46.3
11(RT066)	067	066	5	Pos	67	2.5	74 (16–98)	36	53.7
16	029/163	050	1	Neg	61	2.3	74 (17–94)	33	54.1
36	011/087	011	1	Neg	56	2.1	74 (21–90)	32	57.1
17	005	018	1	Neg	54	2	74 (21–92)	31	57.4
42	062	106	1	Neg	52	1.9	71.5 (3–94)	29	55.8
9	028	081	1	Neg	40	1.5	70.5 (2–91)	23	57.5
34/58	056	056/446	1	Neg	28	1	70 (3–93)	14	50
12	003/293	003	1	Neg	27	1	69 (2–88)	13	48.1
37	017	017	4	Neg	26	1	70.5 (12–93)	13	50

*cdtAB*: genes encoding *C. difficile* binary toxin; Pos: positive presence of *cdtAB*; Neg: negative to the presence of *cdtAB*; RT: PCR ribotype; ST: sequence type; TRST: tandem repeat sequence type

Association of ST, TRST, RT and clades observed in present study and from [14,44-47].

Common types are separated by a slash, while minor types observed in less than 2% are listed in square brackets and left out of the text for simplicity.

### Patients with multiple isolates

In total, 64 patients (2.5%) (129 isolates) carried two or three different types within an 8-weeks time period (Supplementary Table S6). Among these isolates, 41 (32.0%), 18 (14.1%) and 11 (8.6%) were ST1, ST2/13 and ST8, respectively. The time between sampling varied from 0 to 20 days with a median of one day. ST1 and ST2/13 were isolated from 12 patients and ST1 and ST8 from seven patients.

From 39 patients (1.5%) (81 isolates) the same type was isolated more than 8 weeks apart (Supplementary Table S7). Most common isolates were ST2/13 (n = 11, 13.6%) and ST11(RT078) and ST1 (both n = 5, 6.2%). The median time span between sampling was 192 days (range 111–1,286 days). From 32 patients (1.3%) (64 isolates), different types were isolated more than 8 weeks apart (Supplementary Table S8). The median time span between sampling was 185 days (range 107–1,309 days). Most common isolates were ST2/13 (n = 13, 20.3%), ST6 (n = 6, 9.4%) and ST8 (n = 4, 6.3%).

### Temporal analysis

**Figure 1:**
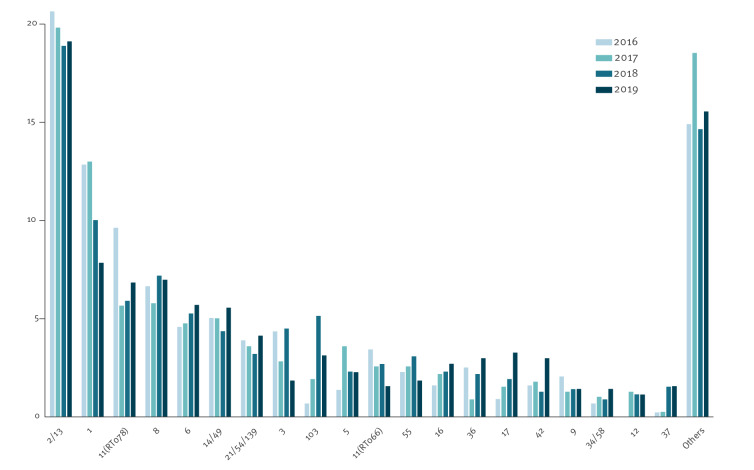
The twenty most common *Clostridioides difficile* ST types listed left to right in order of prevalence by year for the whole country, Denmark, 2016–2019

The 20 most common STs (2,260/2,692, 84.0%) during each of the 4 years are shown in Figure 1. ST2/13 was the major type in all 4 years, ranging from 20.6% in 2016 to 18.9% in 2018. The second most common type was ST1 declining from 12.8% in 2016 to 7.8% in 2019 (p = 0.0008). Other major temporal changes 2016–2019 included an increase of ST103 from 0.7 to 3.1% (p = 0.002), ST17 from 0.9 to 3.3% (p = 0.004) and ST37 from 0.2 to 1.6% (p = 0.003). These temporal changes align with the overall increase in binary toxin-negative isolates, i.e. rising from 70% to 79.5%.

### Geographical distribution of STs in Denmark

**Figure 2:**
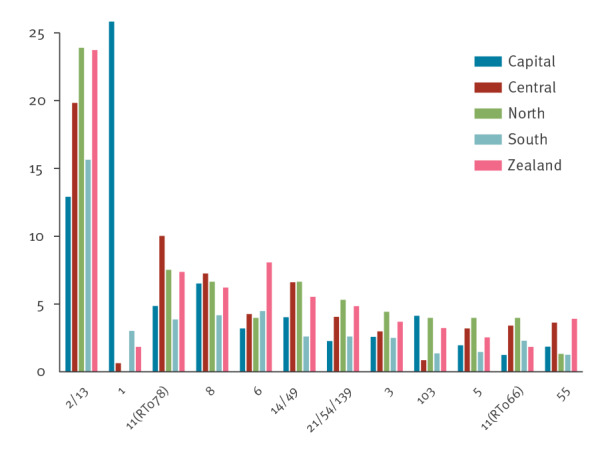
The twelve most prevalent *Clostridioides difficile* ST types and their geographical distribution in per cent within the five regions of Denmark, 2016–2019

Figure 2 shows the 12 most prevalent types identified among the five regions. ST1 was the major *C. difficile* type in the Capital Region (25.8%), whereas ST2/13 was the major type in the remaining four regions (ranging from 12.9 to 23.9%). Most types showed large geographical differences, being most pronounced for ST1. The Simpson’s diversity index based on the type distributions was calculated for each of the five regions for each of the 4 years (Table 1 and Supplementary Table S9). The diversity was higher in 2018–2019 (based on ST derived from WGS) compared with 2016–2017 (based on TRST).

### Children under 2 years

Children under 2 years of age (n = 76) were excluded from the dataset. Their samples predominantly harboured ST2/13 (43.4%), followed by ST8 and ST42 (both 6.6%). Only four isolates (one ST5 and three ST11) harboured the binary toxin genes (Supplementary Table S4).

## Discussion

The present sentinel surveillance system enabled us to describe the demographics of cases and to detect regional and temporal differences, where ST2/13(RT014/020) was the most common type in all regions, with the exception of ST1 in the Capital Region of Denmark. The type distribution of the present sentinel surveillance showed that 75% of all strains lacked the binary toxin gene and 80% (16/20) of the most common types were also binary toxin-negative, underlining the predominance of these strains. Additionally, the ratio of binary toxin-negative strains increased during 2017–2019 (Table 1), indicating a trend where these types have become more common. Females were overrepresented in all of the 4 years, particularly in 2018 for unknown reasons.

Most prominent among binary toxin-negative strains were ST2/13(RT014/020), ST8(RT002), ST6(RT005/117), ST14/49(RT014), ST12/54/139(RT012) and ST3(RT001), consistent with several studies from Europe [18-21], Sweden [22,23], Scotland [24], the US [25-27], worldwide [28] and Australia [29]. ST2/13(RT014/020) accounted for 19.5% of all isolates, and several other studies have found this type predominant and often associated with a community acquired origin [27,29]. A recent Danish study found this type common among dog faeces collected from rubbish bins in public parks in Copenhagen, and closely related (cgMLST) to human clinical isolates collected from the same area during the same time period [30].

ST1(RT027) spread throughout many European countries, including Denmark, after its introduction in the early 2000s [4,9]. The highest numbers were observed in Denmark in 2011–13. However, due to the national surveillance programme targeting this particular type, and subsequent improved local infection prevention measures and control standards such as the introduction of bleach, non-touch room disinfection, isolation of suspected infected patients, antibiotic stewardship (restricted use of fluoroquinolones and cephalosporins) and the use of PCR to detect ST1(RT027) markers, national case numbers have declined over the last 8 years (data not shown). However, despite this reduction, this type still demonstrated endemic spread at hospitals in the Capital Region and at one hospital in the South Region.

ST11 contained two distinct subtypes determined by RT and TRST, namely, RT078/TR070 and RT066/TR067 respectively, here referred to as ST11(RT078) and ST11(RT066). ST11(RT078) has been a predominant, worldwide clone, often described as hypervirulent as ST1(RT027), community acquired, of zoonotic/porcine origin and affecting patients younger than the ST1(RT027)-positive patients [31,32]. Here, ST11(RT078) was the third most common type accounting for 6.7%, with a median patient age of 71.5 years, considerably lower than the median age of 77 years seen for ST1(RT027).

The prevalence of ST103(RT043) increased in the current study from 0.7% in 2016 to 5.1% in 2018. This type has previously been reported in a study from Asia [33]. The geographical distribution showed that half (n = 40) of the isolates originated in the Capital Region, but ST103(RT043) was also relatively prevalent in the North and Zealand Regions. The reason for this clone’s increase is at present unknown, but interestingly, patients infected by this type had the same median patient age as those with ST1(RT027), of 77 years.

ST17(RT018) increased in the present study from 0.9% in 2016 to 3.3% in 2019. This type has previously been found as a predominant type associated with hospital acquired infections in southern Europe, mainly Italy [11], and in Japan [34]. ST17(RT018) has been proposed to have increased mortality due to the production of more toxins and adhesion molecules, in addition to being MDR and having an increased prevalence in elderly patients.

ST5(RT023) was the only type belonging to clade 3 among the 20 most common strains, the third most common type with the binary toxin, and accounted for 2.5% of all *C. difficile* infections in the present study. It has been linked to severe clinical outcome comparable to ST1(RT027) and ST11(RT078), and the prevalence is consistent with previous studies [5,35].

ST37(RT017) is one of a few types only producing TcdB, and has been associated with several outbreaks around the world and considered endemic in parts of Asia. Some reports have shown higher mortality and resistance towards several antibiotics [12,36]. ST37(RT017) found in Denmark in 2018–19 through the sentinel surveillance, did have a 30-day overall mortality of 32% and was genotypic resistant towards fluoroquinolones, aminoglycosides, tetracycline, macrolides and rifampicin [37]. The prevalence of ST37(RT017) was found to increase from 0.2% (2016) to 1.6% (2019) in the present study, and as such is now being followed closely through the national surveillance programme.

Children younger than 2 years are usually not considered prone to CDI, mainly due to immature toxin receptors in the gut [38] and therefore, this age group was excluded from the present dataset. Still, this age group can carry *C. difficile* and studies have found RT014/020 to be common among children under 2 years [39,40]. This phenomenon was in agreement with the present study where we reported 43% of all *C. difficile* strains in this age group to be ST2/13(RT014/020).

In 2013, we changed typing method from PCR ribotyping to TRST because TRST generated easy and exact interpretation from Sanger sequencing of the two tandem-repeat regions. In 2018, we changed to WGS as this had become the predominant method in our department and facilitated new and more detailed typing analysis. Therefore, we changed the typing scheme to 7-locus MLST, as this is easily extracted from the WGS data and allows direct comparison to international studies using the same nomenclature. Regarding backward traceability, some types were difficult to compare between the three typing methods because: (i) they all have unique resolution; (ii) they do not always translate one to one; (iii) new types are constantly evolving and (iv) corresponding types are sometimes reported differently. Therefore, the associations listed in Table 2 are not to be considered ubiquitous, but are what we observed from our analysis and what has been reported in the literature.

This study was not designed to investigate reinfections and recurrences, as we only included samples during two fixed periods per year. Still, a number of patients participated in the study with more than one isolate and a number of observations can be made. Among patients with different types less than 8 weeks apart there was an overrepresentation of ST1(RT027) (32.0%). All of those originated from one particular DCM in the ST1(RT027)-endemic area of the Capital Region and is therefore attributed to the higher local prevalence of ST1 and a more extensive test strategy for *C. difficile*. Among patients with an identical type of *C. difficile* more than 8 weeks apart, often associated with relapse infections, the type distribution resembled the general dataset with major types being ST2/13(RT014/020), ST11(RT078) and ST1(RT027) as also observed by [23,41,42]. Among patients with different types more than 8 weeks apart, indicating reinfections, the types were dominated by the binary toxin-negative strains ST2/13(RT014/020), ST6((RT005/117) and ST8(RT002), also common in the general dataset.

The present sentinel surveillance has some limitations: (i) loss of resolution when typing was obtained from two different methods; (ii) prevalence of low abundant types and Simpson’s diversity index applied on two different typing methods must be interpreted with caution; (iii) data were only obtained from limited sentinel periods, missing out seasonal and other changes between the periods, including reduced continuity to follow individual patients with respect to multiple episodes; (iv) bias from individual DCMs using different patient inclusion criteria, i.e. no strict case definition as recommended by the ECDC [13]; (v) bias from individual DCMs using different diagnostic methods and sample collection and no DCM used the internationally recommended 2-step algorithm [43]. Still, a toxigenic *C. difficile* isolate was recovered from 89% of the samples received during the study, where most DCMs used PCR as the primary method, and others have argued that PCR diagnostics are suitable for surveillance purposes [29].

### Conclusion

The sentinel surveillance presented here has been implemented as a routine part of the national surveillance programme for *C. difficile*. The present setup is a valuable tool as all 10 Danish DCMs participate, i.e. the entire country is included and it offers a more accurate and unbiased prevalence study of types, including binary toxin-negative strains, and visualises the temporal and geographical changes within 6-month periods. The production of WGS data on all isolates was initiated in 2018 in the national and sentinel surveillance programmes, and is of course costly, but allows for further characterisation of isolates for surveillance, as well for research without additional laboratory work. Currently, cgMLST is being used to investigate possible outbreaks and transmission events and the detection of relevant markers of antimicrobial resistance is under validation and will be included in our national surveillance in the near future.

## Supplementary Data

152000 Supplementary Material
